# Molecular detection of virulence factors and biofilm formation in *Pseudomonas aeruginosa* obtained from different clinical specimens in Bandar Abbas

**Published:** 2019-02

**Authors:** Nima Bahador, Saeed Shoja, Foroogh Faridi, Banafsheh Dozandeh-Mobarrez, Fatemeh Izadpanah Qeshmi, Sedigheh Javadpour, Sedigheh Mokhtary

**Affiliations:** 1 Infectious and Tropical Diseases Research Center, Hormozgan Health Institute, Hormozgan University of Medical Sciences, Bandar Abbas, Iran; 2 Department of Microbiology, College of Sciences, Agriculture and Modern Technology, Shiraz Branch, Islamic Azad University, Shiraz, Iran

**Keywords:** *Pseudomonas aeruginosa*, Virulence factors, Biofilm

## Abstract

**Background and Objectives:**

*Pseudomonas aeruginosa* is a ubiquitous opportunistic pathogen. The presence of several virulence factors such as exotoxin and exoenzyme genes and biofilm may contribute to its pathogenicity. The purpose of this study was to investigate the presence of *toxA, exoU* and *exoS*, the determination of biofilm production and antimicrobial susceptibility patterns among clinical isolates of *P. aeruginosa*.

**Materials and Methods:**

In this study, 75 isolates of *P. aeruginosa* were recovered from various clinical specimens. Antimicrobial susceptibility pattern of isolates were identified. Virulence genes *toxA, exoU* and *exoS* were determined using PCR. The ability of biofilm production was assessed.

**Results:**

Antimicrobial susceptibility test showed that 12 strains were resistant to more than 8 antibiotics (17.14%). The most effective antibiotic was colistin as 98.6% of isolates were sensitive. The frequencies of *exoU* and *exoS* genes were detected as 36.6% and 55.7%, respectively. In addition, 98.6% of the isolates were biofilm producers. Exotoxin A was detected in sixty-eight isolates (95.7%).

**Conclusion:**

The findings of this study showed that, the presence of *P. aeruginosa* exotoxin and exoenzyme genes, particularly, the *exoU* gene is the most common virulence factors in the bacterial isolates from urine samples. Biofilm is a serious challenge in the treatment of *P. aeruginosa* infection.

## INTRODUCTION

*Pseudomonas aeruginosa* has emerged as an important nosocomial infection. This bacterium causes infection especially in the hosts with compromised defense mechanisms such as patients with severe burns and individuals with HIV infection (1–4). It causes a wide range of infections including septicemia, pneumonia, endocarditis, burn wounds, otitis and keratitis (5, 6). *P. aeruginosa* is responsible for mortality rates as high as 50%. It may quickly become problematic once introduced in a hospital because of its ability to adhere to medical devices such as catheters (7). This organism is often resistant to antibiotics and enters the blood, causing septicemia (8). *P. aeruginosa* appears to be related to the production of a large number of secretions and cell-associated virulence factors including toxins, enzymes and biofilm (9). Growth in biofilm promotes bacterial survival. Once a biofilm is formed, it becomes extremely difficult to be destroyed (10). Biofilm is a complex aggregation of microorganisms in an exopolysaccharide matrix and is usually resistant to antibiotics (9). *P. aeruginosa* is a pathogen with innate resistance to many antibiotic classes and these include aminoglycosides, carbapenems, antipseudomonal penicillins, quinolones and cephalosporins. In addition, it has been known to acquire novel resistance genes via horizontal gene transfer (2, 11, 12). *P. aeruginosa* also possesses a variety of virulence factors such as exotoxin A (encoded by *toxA* gene), exoenzyme S (encoded by *exoS* gene), exoenzyme U (encoded by *exoU* gene). *ExoA* is the major constituent of the type II secretion system (T2SS) which inhibits protein synthesis through the transfer of the adenosine diphosphate-ribosyl moiety from nicotinamide-adenine dinucleotide to elongation factor 2, resulting in the inhibition of protein. Another important virulence factor that was recently recognized is the type III secretion system (T3SS). T3SS is a contact-dependent protein secretion pathway that plays a major role in the pathogenesis of serious *P. aeruginosa* infections. This system secretes effector proteins such as *ExoS* and *ExoU* (12). *ExoS* is a major cytotoxin that is required for colonization, invasion and bacterial dissemination during infection (2, 7, 13). *ExoU* is a cytotoxin with phospholipase activity that affects epithelial cells and causes lung infection (14). In addition, *exoU* has a toxic effect on macrophages (11, 12). One of the most important virulence determinants of this bacterium is the biofilm which is sessile populations of microorganisms that are enclosed by the self-secreted extracellular polysaccharide matrix or slime layer. Biofilms act as efficient barriers against antimicrobial agents (4). Multidrug resistant forms of *P. aeruginosa* (MDRPA) are a major source of nosocomial infections (15). The increasing resistance of *P. aeruginosa* to numerous antibiotics, because of excessive antibiotic administration, is now leading to the accumulation of antibiotic resistance and cross-resistance between antibiotics and the appearance of multidrug-resistant (MDR) forms of *P. aeruginosa* (16). It has been shown in previous studies that MDR strains are widespread among Iranian hospitals (2). The purpose of this study was to investigate the presence of *toxA, exoU* and *exoS*, the determination of biofilm production and antimicrobial susceptibility patterns among *P. aeruginosa* in Bandar Abbas, southern part city of Iran.

## MATERIALS AND METHODS

### Bacterial isolates and identification test.

In this cross-sectional study, undertaken from April 2017 to July 2017, 75 non-duplicates isolates of *P. aeruginosa* were obtained from patients admitted to Shahid Mohammadi and Pediatrics Hospitals at Bandar Abbas. Bacterial isolates were collected from different clinical samples such as wounds, respiratory tract, urine, blood, sputum, eye and CSF. Each isolate was cultured in MacConkey agar and Blood agar and plates were incubated at 37°C overnight. Isolates were identified using biochemical tests (8). All bacterial isolates were stored in a micro tube containing tryptic soy broth (TSB) with 20% glycerol at −70°C until further investigation (8, 14).

### Antibiotic susceptibility tests.

Antimicrobial susceptibility of the isolates was determined by the Kirby-Bauer disk diffusion method on Muller-Hinton (Merck, Germany) agar according to Clinical and Laboratory Standards Institute (CLSI) guideline (17). Briefly, a suspension of each isolates was adjusted to a turbidity equivalent to 0.5 McFarland and inoculated on Muller-Hinton agar plate. The tested antimicrobial agents were as follows: piperacillin (100 μg), ceftazidime (30 μg), meropenem (10 μg), imipenem (10 μg), gentamicin (10 μg), amikacin (30 μg), ofloxacin (30 μg), cefotaxime (30 μg), colistin (10 μg), ciprofloxacin (5 μg), tetracycline (75 μg) and aztreonam (30 μg) (MAST, Group Ltd, Merseyside, UK) (8, 13). *E. coli* ATCC 25922 and *P. aeruginosa* ATCC 27853 were used as standards in disk diffusion. The plates were incubated overnight at 35°C and the results were interpreted as susceptible, intermediate or resistant according to the criteria recommended by the CLSI (2). MDR isolates were defined if they showed simultaneous resistance to one agent from three antibiotics group (18).

### Identification of bacterial isolates as *P. aeruginosa* by PCR.

To approve bacterial isolates as *P. aeruginosa*, all isolates were checked for the presence of *gyrB* gene with specific primers as detiled in Table 1 (19).

**Table 1.** Primers used in this study

**Primer Target**	**Oligonucleotide Sequence (5′-3′)**	**Amplicon Size (bp)**	**Target Gene**	**TM °C**	**References**
*toxA*-F	TGCTGCACTACTCCATGGTC	190	*toxA*	53.8	(24)
*toxA*-R	ATCGGTACCAGCCAGTTCAG			53.8	
*exoU*- F	GCTAAGGCTTGGCGGAATA	204	*exoU*	51.1	(10)
*exoU*-R	AGATCACACCCAGCGGTAAC			53.8	
*exoS*- F	ATGTCAGCGGGATATCGAAC	230	*exoS*	51.1	(10)
*exoS*-R	CAGGCGTACATCCTGTTCCT			53.8	

### Extraction of DNA.

DNA was extracted from the isolates by the modified TE boiling method. Each sample was suspended in 200 μL of TE buffer [10 mM/L Tris-HCl, 1 mM/L EDTA (pH 7.5)]. Each suspension was centrifuged at 8000 rpm for 4 min at 4°C 3 times. The pellet was re-suspended in 200 μL TE buffer, mixed on a vortex mixer and subjected to boiling at 100°C in a heating block (Boeco, Germany) for 1 min. Three freeze-thaw cycles were performed alternating between −70°C for 3 min and 100°C for 2 min. The tubes were centrifuged at 10000 rpm for 5 min. The supernatant was transferred to a sterile tube and stored at −20°C (20).

### Polymerase chain reaction amplifications.

PCR was carried out to detect *toxA, exoU* and *exoS*. The final volume of each reaction was 25 μL, containing 2 μL of DNA, 1× PCR buffer, 2 mM MgCl_2_, 200 μM of dNTP, 0.2 μM each of primer and 1 U Taq polymerase (SinaClon, Iran). PCR amplification for *toxA, exoU* and *exoS* genes were performed according to the following program: initial denaturation at 94°C for 5 min; 30 cycles of denaturation at 94°C for 30 s; annealing of *toxA* at 60°C, *exoU* at 56°C and *exoS* at 58°C for 50 s; extension at 72°C for 50 s; and a final extension step at 72°C for 10 min. For staining of the gel, each PCR product was mixed with 5× Gel Red (Biotium, USA) and then separated on electrophoresis on 1.2% agarose gel in TBE buffer (7.5 g EDTA, 108 g Tris and 55 g boric acid). After electrophoresis, they were visualized under UV transilluminator. One PCR product was sequenced for each gene and then used as a positive control in PCR reactions. Sequences were submitted to the GenBank. Nucleotide sequence database under accession numbers: LT615361 for *toxA*, LT615362 for *exoS* and LT628504 for *exoU*.

### Biofilm formation.

All isolates were analyzed for their abilities to produce biofilm using a colorimetric microtiter method. In order to quantitatively determine biofilm formation capacity, bacterial colonies were grown overnight at 37°C in Tryptic Soy Broth (TSB) (Merck Darmstadt, Germany). The bacterial suspensions were diluted (1:100) in a new TSB medium and 150 μL of this solution was inoculated onto the sterile flat-bottomed 96-well polystyrene microtiter plates. After incubation for 24 h at 37°C, the wells were gently washed three times with distilled water. The wells were dried in an inverted position at room temperature and finally stained with 125 μL of 0.1% crystal violet solution (CV) in water for about 10–15 min. Crystal violet was discarded; and to remove extra CV, the wells were washed three times. Finally, the bounded CV was released by the addition of 125 μL of 30% acetic acid. A new sterile plate was inoculated with 125 μL distaining solutions in each well. The absorbance of optical density (OD) of each well was measured at 550 nm using an ELISA reader (Biotek elx800). All the assays were repeated three times. As a control, the un-inoculated medium was used to determine background OD. The cut-off OD (ODc) was defined as three standard deviations above the mean OD of the negative control. According to the results of the microtiter plate test, the isolates were classified into the following four categories based on the optical density: non-biofilm producers (OD test <ODc), weak biofilm producers (ODc< OD < 2× ODc), moderate biofilm producers (2× ODc< OD < 4X ODc) and strong biofilms producers (4× ODc< OD) (21, 22).

### Statistical analysis.

Statistical Package for Social Sciences (SPSS) software version 23 was used for statistical analyses. The correlation between the prevalence of the virulence gene, antibiotic resistance patterns, and biofilm production were determined using Pearson's Chi-square test. A P value of < 0.05 was considered to be statistically significant.

## RESULTS

The rate of bacteria from each sample was as follows: urine 31.5%, respiratory tract sample 28.5%, wounds 20%, blood 8.6%, eye 7.1% and CSF 4.3%. The resistance pattern to the 12 antimicrobials tested is shown in Table 2. According to the results, a high rate of resistance was observed for tetracycline (32.85%) and ofloxacin (30%). The low resistance was observed for colistin (1.42%). One isolate (1.42%) was resistant to all the tested antibiotics. In addition, 58.6% of the isolates were sensitive to all the antimicrobial agents. In total, 24.3% of the isolates showed resistance to at least three different classes of antimicrobial agents and were identified as MDR. Amplification analysis showed that 95.7, 55.7 and 38.6% of the samples harbored *toxA, exoS* and *exoU*, respectively. The co-existence of *exoS* and *exoU* was detected in 4.28% of the isolates (Table 3). *exoU* and *exoS* genes were significantly prevalent in the urine and wound isolates, respectively. Biofilm data showed that 98.6% of the isolates were biofilm producers, in which, 60% were strongly biofilm producers and the rates of moderate and weak biofilm producers were 34.3 and 4.3%, respectively. However, biofilm formation was not found in 1.4% of the isolates. Interestingly *exoS* and *exoU* positive strains had a significantly higher ability to form biofilm.

**Table 2.** Antimicrobial resistance pattern of *P. aeruginosaisolates*

**Samples**	**Antibiotic**											

	**Gentamicin**	**Imipenem**	**Ceftazidim**	**Aztreonam**	**Ciprofloxacin**	**Amikacin**	**Colistin**	**Oflacxine**	**Tigecyclin**	**Piperacilin**	**Meropenem**	**Tetracyclin**
Urine	2	3	2	2	4	2	0	5	1	1	1	3
Blood	2	2	2	2	2	2	0	2	3	2	2	5
Wound	2	3	2	2	2	2	0	4	0	0	3	3
Respiratory	3	5	8	4	3	2	0	7	6	3	5	9
Eye	0	0	0	0	0	0	0	0	0	0	1	0
CSF	3	3	3	3	3	3	1	3	1	3	3	3
Total	12	16	17	13	14	11	1	21	11	9	15	23
(%)	(17.14)	(22.85)	(24.28)	(18.57)	(20)	(15.71)	(1.42)	(30)	(15.71)	(12.85)	(21.42)	(32.85)

**Table 3.** Frequency of exoenzymegenes

	***exoS***		**Total**

	**Yes**	**No**	
*exoU* Yes	3 (4.28)	24 (34.28)	27 (38.57)
*exoU* No	36 (51.42)	7 (10)	43 (61.42)
Total	39 (55.71)	31 (44.28)	70 (100)

## DISCUSSION

*P. aeruginosa* is an important opportunistic cause of nosocomial infections and it has developed resistance to ranges of antimicrobial agents in immunocompromised patients (1, 2). In *P. aeruginosa* infections, biofilm production has been observed as an important determinant of pathogenicity. The formation of biofilms facilitates chronic bacterial infections and reduces the efficacy of antimicrobial therapy (1, 13). According to the results of this study, 98.6% of the *P. aeruginosa* isolates produced biofilms, and among them, 60% were strong biofilm producers. Our results showed 68% of isolates with strong biofilm were resistant to tetracycline. In a study by Ghanbarzadeh et al. and Jabalameli et al. respectively, in Iran, 92.4 and 96.9% of the isolates produced biofilm, which are in accordance with the results of this study (2, 9). It is noteworthy that the rate of strong biofilm production in urine samples (65%) was higher than other samples. The study of the relationship between biofilm formation and antibiotic resistance showed that MDRPA isolates produced strong biofilm. In recent years, several reports confirmed an increasing multidrug resistance among *P. aeruginosa* isolates from nosocomial infections in Iranian hospitals (15). In this study, only 1.4% of the isolates were resistant to colistin. This finding showed that this antibiotic could be used in first line for the treatment of infections due to *P. aeruginosa.* Unfortunately, colistin is the last choice of therapy for these infections. Recently, colistin resistance to *P. aeruginosa* has been reported from different parts of the world. Interestingly, 58.6% of the total isolates were sensitive to all antibiotic agents. In contrast to other studies that showed a high prevalence of antibiotic resistance, in this study, lower prevalence of antibiotic resistance was observed. *P. aeruginosa* secretes effector proteins such as *exoS, exoU* and *toxA.* In isolates with strong biofilm, 58.82%, 32.35% and 29.41% of them were positive for *toxA, exoS* and *exoU* genes, respectively. These proteins modify host cell functions, which are important in cytoskeletal organization and signal transduction. *ExoS* is a bi-functional toxin exhibiting ADP-ribosyltransferase and GTPase-activating activity. *ExoU* exhibits phospholipase activity and disrupts eukaryotic membranes following its delivery into the cytoplasm. In our investigation, 100% of the *P. aeruginosa* isolates from the eye and the wound samples were *toxA*+, which is the same as Yousefi-Avarvandet al.'s study (14). *ToxA* was the most frequent (95.7%), which is higher than Ghanbarzadeh's (75%) and Amirmozafari's (81%) studies (2, 13). In a study from Iran, Yousefi-avarvand reported 65.4 and 66.7% for *exoU* and *exoS*, respectively, and this is higher than that of the current study (14). Firouzi-Dalvand and Pooladi reported the rates of *exoU* (22%) and *exoS* (14%) in burn samples, so, their findings for the genes are less than those of the current study (10). In Poland, Pobiega reported a rate for *exoU* (19.2%) and *exoS* (92.3%) (12). In a research in Iran by Heidary, the frequency of *exoS* and *exoU* among the clinical isolates of *P. aeruginosa* was 92.95 and 56.33%, respectively, which is in contrast to this study. However, the frequency of *exoU* in the current study is comparable to that of Habibi and Honarmand's study (37.5%) (23).

**Fig 1.**
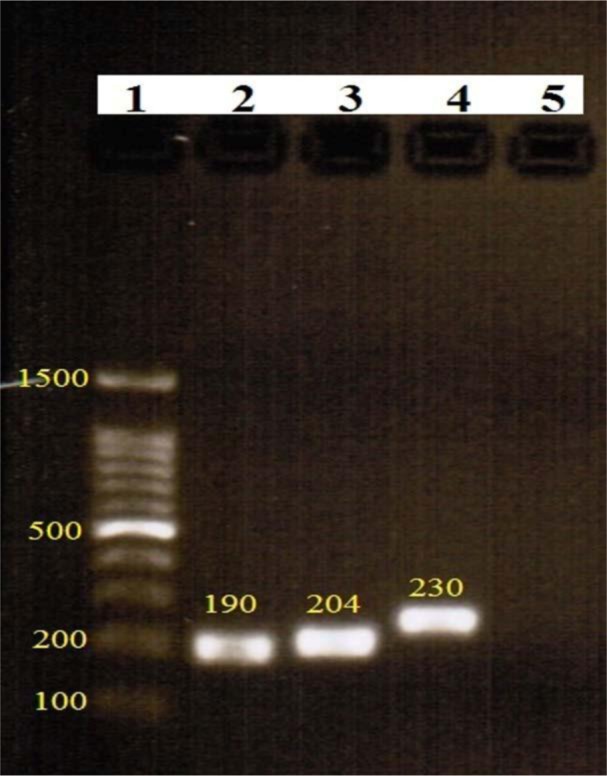
Electrophoresis results of virulence genes PCR Lane 1: ladder 100 bp, lane 2: *toxA*, lane 3: *exoU*, lane 4: *exoS* and lane 5: negative control

## CONCLUSION

In conclusion, this study showed that the exotoxin A and exoenzyme S and U genes are commonly disseminated among the *P. aeruginosa* isolates from patients bed fast in hospitals. Moreover, a high rate of biofilm producer isolates were found in urine samples, which is a serious problem in the treatment of urinary tract infections. Additional studies are necessary on other virulence factors to obtain more information about *P. aeruginosa*, in order to find a way to diagnose and treat infections more quickly.
